# Temporal Preparation for Speaking in Question-Answer Sequences

**DOI:** 10.3389/fpsyg.2017.00211

**Published:** 2017-02-21

**Authors:** Lilla Magyari, Jan P. De Ruiter, Stephen C. Levinson

**Affiliations:** ^1^Department of General Psychology, Faculty of Humanities and Social Sciences, Pázmány Péter Catholic UniversityBudapest, Hungary; ^2^Department of Psychology, School of Arts and Sciences, Tufts University, MedfordMA, USA; ^3^Language and Cognition Department, Max Planck Institute for PsycholinguisticsNijmegen, Netherlands; ^4^Donders Institute for Brain, Cognition and Behaviour, Radboud UniversityNijmegen, Netherlands

**Keywords:** turn-taking, timing, preparation, speech production, anticipation

## Abstract

In every-day conversations, the gap between turns of conversational partners is most frequently between 0 and 200 ms. We were interested how speakers achieve such fast transitions. We designed an experiment in which participants listened to pre-recorded questions about images presented on a screen and were asked to answer these questions. We tested whether speakers already prepare their answers while they listen to questions and whether they can prepare for the time of articulation by anticipating when questions end. In the experiment, it was possible to guess the answer at the beginning of the questions in half of the experimental trials. We also manipulated whether it was possible to predict the length of the last word of the questions. The results suggest when listeners know the answer early they start speech production already during the questions. Speakers can also time when to speak by predicting the duration of turns. These temporal predictions can be based on the length of anticipated words and on the overall probability of turn durations.

## Introduction

Recently, there has been increasing interest in interactive language processing in psycholinguistics (e.g., Barr, 2008; Branigan et al., 2010; Willems et al., 2010; Bašnáková et al., 2015; Garrod and Pickering, 2015; Levinson and Torreira, 2015). For a long time, experimental studies of language processing have focused mainly on how individuals comprehend or produce phonemes, words and sentences, while the social setting in which language is used has rarely been investigated. In an every-day conversation, participants also need to coordinate their turns in content and need to time their production. Hence, if we want to study human language capacity an important issue is how an interactional context influences language processing.

One striking aspect of natural conversations is the give and take of turns between conversational partners. A systematic way of turn-taking appears early during human development and it precedes the development of linguistic competence. Moreover, it has also been suggested that turn-taking appeared prior to language in phylogeny (Levinson, 2016). During conversational turn-taking, speakers and listeners alternate freely, without the restrictions of any institutional settings (e.g., interactions between teacher and student or between doctor and patient; Levinson, 1983). Observations have shown that turn-transitions of natural conversations –the switches between the speaker’s and the listener’s roles– happen remarkably quickly. For example, 45% of the turn-transitions are between +250 and -250 ms in Dutch telephone-conversations (De Ruiter et al., 2006). Experimental studies have estimated how long the process leading to the initiation of word- and sentence-production takes. This duration is longer than the most frequent turn-transition times of conversations. In picture-naming studies, it takes at least 600 ms to name an object (Levelt, 1989; Indefrey and Levelt, 2004; Indefrey, 2011). Naming times are not radically shorter when words have a higher frequency or when they are repeated during an experiment (Jescheniak and Levelt, 1994; Damian et al., 2001). In contrast, turn-transition durations are often shorter than 200 ms in conversations in several languages (Stivers et al., 2009). Given the latency of the speech production process, it seems listeners (who are next to speak) often cannot wait until the current speaker finishes, but must begin the production process prior to the end of the current turn (Levinson, 2013).

Therefore, listeners are probably required to execute parallel cognitive tasks for an immediate response. They need to understand the message of the current turn and start to prepare an answer. The short transitions also suggest that next speakers time the production of the answer to the end of the current turn.

In order to prepare an answer before the current turn ends, listeners must know what they want to say. Therefore, it is likely that they anticipate the message of the turn they are listening to. Studies using eye-tracking and electrophysiological (EEG) measurements have shown that people anticipate information at many different levels of language processing. Eye-tracking studies have demonstrated semantic predictions made by listeners (e.g., Kamide et al., 2003; Knoeferle et al., 2005; Altmann and Kamide, 2007). Event-related potential studies have showed syntactic (Wicha et al., 2004; Van Berkum et al., 2005) and word-form (DeLong et al., 2005, 2011) predictions. EEG studies have also showed that lexical items or their syntactic and semantic features were pre-activated before the predicted item has been heard or seen (DeLong et al., 2005, 2011). Kutas et al. (2011) conclude that the predicted and not yet heard linguistic input can already alter information processing. Regarding conversations, this suggests that it is possible to start the preparation of an answer before the turn end, as soon as the content of the turn can be anticipated.

Regarding the timing of a response, the tight transition-times also suggest that next speakers wait for the right moment to deliver the answer (De Ruiter et al., 2006). A timed production is only possible if next speakers know in advance when the current turn will be completed. Different mechanisms have been suggested to explain how people know when a turn will end. For example, Sacks et al. (1974) suggest that sentential, clausal, phrasal, and lexical constructions can project when a turn will be completed. Although they have left open the question how “projection” is accomplished, their account suggests that lexical and syntactic anticipations provide the necessary information for turn-end predictions.

Others have argued that timed production of turns is not a result of anticipation. This view holds that turn endings are signaled to the listener through a variety of means, including eye-gaze (Kendon, 1967; Duncan, 1974) and changes in prosody (Duncan, 1974; Duncan and Fiske, 1977; Beattie et al., 1982; Schaffer, 1983; Local et al., 1985, 1986; Cutler and Pearson, 1986; Local and Walker, 2012). Regarding eye-gaze, later studies have shown that turn-transition times are not longer during telephone conversations than in face-to-face interaction (Ten Bosch et al., 2005; De Ruiter et al., 2006; Stivers et al., 2009), and that in general, gaze-behavior is also influenced by the social actions and goals of the participants and not only by the organization of the turns (Rossano et al., 2009; Rossano, 2012). Therefore, eye-gaze cannot play a systematic role as a turn-transition cue. With respect to prosody, most of the studies have focused on the role of pitch in signaling turn-ends (for an overview see De Ruiter et al., 2006; Local and Walker, 2012). A few experimental studies tried to evaluate the relative contribution of the different information sources, e.g., intonation in the prediction of turn-ends (Beattie et al., 1982; Schaffer, 1983; Cutler and Pearson, 1986; De Ruiter et al., 2006; Casillas and Frank, 2013; Bögels and Torreira, 2015). Cutler and Pearson (1986) recorded dialogs by having speakers read written scripts. Fragments of these dialog-recordings were given to participants who had to indicate whether the fragments were turn-medial or turn-final. They found that falling intonation contour indicated turn finality while rising intonation served as a turn-yielding cue, but they noted that many of the utterances which were found ambiguous by the listeners also had falling or rising pitch. In a recent study, Bögels and Torreira (2015) found that intonational phrase boundaries helped listeners to decide whether a turn ended or continued. However, De Ruiter et al. (2006) found that accurate turn-end prediction was possible without pitch information and intonation-contour. In their experiment, participants listened to turns taken out of conversational context. The stimuli used in the experiment were from recordings of natural conversations. The task of the participants was to press a button exactly when each turn ended. The button-presses were not less accurate for turns from which the intonation contour was removed compared to the original recordings. But the button-presses were significantly worse when the recordings were manipulated in such way that the intonation was intact but words could not be understood. De Ruiter et al. (2006) have concluded that lexical and syntactic information plays a major role in turn-end prediction, and not intonation.

However, it remained an open issue how syntactic and lexical information facilitates turn-end predictions. One possibility is that the syntactic-semantic content of turns informs the listener whether the currently heard linguistic element will terminate the turn or if it will be followed by further information. In this case, the terminal element serves as a cue for response initiation. Speakers do not have expectations of when the turn end will occur, but they will start speech production when they recognize the terminal element (e.g., the last word) of the turn. This account is also compatible with some studies which have investigated when speakers begin speech planning while listening to their conversational partner. For instance, Sjerps and Meyer (2015) employed a dual task paradigm using a finger tapping task. In one of the experimental conditions, participants were taking-turns with pre-recorded speech while they were tapping a complex pattern with their fingers. The tapping performance was disrupted only shortly before the end of the previous turn. The disruption was interpreted as a sign that the cognitively demanding speech planning started. Sjerps and Meyer (2015) concluded that speech planning overlaps with the previous utterance and it starts only quite late time-locked to the turn-end. However, another study (Bögels et al., 2015) have found evidence using EEG measurement during an interactive quiz-paradigm that production planning of answers starts as soon as possible during questions. In this study, the production process started at least in a half second after the answer to a question could be guessed which could happen up to several seconds before the end of the questions. The finding of this study is not incompatible with the use of terminal elements as a cue for response initiation. Several studies have shown that the speech production process involves several stages from conceptual preparation to lexical access, phonological processing and articulation (Dell, 1986; Levelt, 1989; Caramazza, 1997). The findings of Bögels et al. (2015) suggest that speakers initiated some part of the speech planning processes much earlier than the terminal elements of turns and they delayed the articulation process until the end of the question. Therefore, it is possible that speakers started the articulation process when they encountered a turn-ending cue.

The findings of these two studies (Bögels et al., 2015; Sjerps and Meyer, 2015) have been inconsistent about when speakers start speech planning in situation of turn-taking. However, their findings can be reconciled. Studies have shown that speakers plan their utterances incrementally, i.e., they do not plan the entire utterance before articulation (e.g., Bock and Levelt, 1994; Griffin, 2001) and that the size of the planning units can be flexible (Ferreira and Swets, 2002; Konopka, 2012; Swets et al., 2014). Ferreira and Swets (2002) also showed that the duration of utterance planning can be affected by external task demands (e.g., time pressure). Therefore, the onset of speech planning might vary across situations depending on several factors (e.g., working memory, linguistic complexity, time-pressure). It is an interesting issue which factors might influence and how they could influence the onset of speech planning. One of the research questions of this study was whether the time when speakers could guess the answer to questions affects speech planning. We hypothesized that if answers can be guessed only at the recognition of the last word of questions, this might result in a delay. When answers can be guessed much earlier, speaker might use the time and start the initial stages of speech planning before the last word, so they can produce responses closer to the end of turn.

Even if the initial stages of speech planning could start early, the question still remains how speakers time the articulation of their turn. We have already presented several studies which have suggested that speakers start articulation (or speech planning) as a reaction to cues in their partner’s turn (see Local and Walker, 2012). These cues have been mainly described in the prosody of the speaker’s turn. We have also pointed out that lexical information, e.g., the recognition of the last word of a question could also serve as a cue to initiate a response. However, other mechanisms for the precise timing of turn-transitions might also exist. It is also possible that listeners predict the duration of turns, and therefore, they prepare for the articulation relative to how late they predict the turn-end coming. Anticipated syntactic structure and words are good candidates for providing information about the duration of the rest of the turn. According to Pickering and Garrod’s language production and comprehension account (Pickering and Garrod, 2013), speakers are able to predict the content and also the timing of their interlocutor’s utterance. They argue that listeners use simulation with the help of the language production system to predict the content of utterances. This prediction, i.e., a forward model also represents time. For example, if up-coming words are predicted, listeners could predict when the articulation of the given and the next word will start. According to this account, the forward models are likely to be “impoverished” in the sense that they do not contain information about how phonemes are produced, or information about the entire phonological form of predicted words. De Ruiter and Cummins (2013), however, argue that temporal predictions based on “impoverished” representations cannot explain the temporal accuracy observed in end-of-turn predictions.

In another study, Garrod and Pickering (2015) suggest that listeners use oscillatory entrainment to predict the precise timing of their interlocutor’s turn. The predicted linguistic representations are combined with the interlocutor’s speech rate to which the listener’s brain circuitry is entrained by low-level acoustic analysis. According to their model, speakers will determine the appropriate timing for turn transitions using these predictions.

However, there is still little empirical evidence that listeners can make predictions about the duration of linguistic information and whether these temporal predictions influence turn-transitions. A related open issue is the span of such temporal predictions. Listeners might predict the durations of the final syllable of turns by entrainment to the speakers’ speech rate. They might also be able to make predictions about the duration of longer segments, e.g., several syllables or words. To address this issue, Magyari and De Ruiter (2012) studied whether anticipation of the last words of turns, and the number of words of turns correlates with well-timed turn-end predictions. Their experiment used a subset of turns from De Ruiter et al.’s (2006) previous experiment. Recordings of the turns were truncated at several points before the end. Participants listened to the segments of the turns and guessed how the turns end. Guesses about the last words were more accurate when initial segments of those turns which had more accurate button-presses in the previous experiment were presented. The number of the guessed words also correlated with the button-presses. Segments of turns with later button-presses were associated with a larger number of guessed words than the actual number of words in the continuations. In another study, a neuronal correlate of turn-end prediction revealed that predictions concerning the turn end started more than a second before the end of turns when the content of the turns could be predicted (Magyari et al., 2014). The findings of these studies suggest that anticipation of the content of turns provides temporal information about the turn-end before the last word of turns. In these studies, however, turn-end predictions were measured by using a button-press paradigm. It has been rarely shown by experimental studies that temporal linguistic predictions affect verbal response times, i.e., turn-transitions.

The “reaction on a cue” and the “temporal preparation” accounts are not necessarily incompatible with each other. When next speakers are already prepared for the motor response, they will respond faster when they encounter a turn-ending cue or the turn-end. They can also inhibit the execution of the articulatory movements by attending to cues which signal that the turn is not ending yet. Such “talk-projecting” cues have already been described in intonation and in non-pitch phonetic features of words (Caspers, 2003; Local and Walker, 2012).

Most of the studies of turn-taking presented thus far have focused on the different features of the linguistic content of turns (e.g., intonation, syntactic and lexical predictions, speech rate) which can help listeners, i.e., next speakers of a conversation in timing their turns. However, little attention has been paid to why estimation of turn durations could lead to better timed turn-transitions. Previous research on temporal preparation has shown that participants not only wait for a signal to react on but they prepare for the moment when the signal is most probably going to appear. These studies have usually employed non-verbal tasks where the time intervals between a warning signal and an imperative response signal have been manipulated. Several experiments have shown that reaction times to a response signal will decrease if participants can estimate the point in time when the response signal is delivered (relative to the warning signal). Reaction times decrease when the occurrence of the response signal can be estimated, because participants can prepare for their response (Niemi and Näätänen, 1981; Trillenberg et al., 2000; Müller-Gethmann et al., 2003). Moreover, the level of response preparation is proportional to the subjective probability of the occurrence of the response signal at any given moment. For example, Trillenberg et al. (2000) showed that response times were affected by the probability with which the warning signal was presented at each time-point in the experiment. For verbal interactions, these findings suggest when speakers are certain about when the other’s turn is going to finish, they will be more prepared for the articulation, hence, speakers’ response latencies will decrease. Moreover, interactants might take into account how long the other’s turns usually are which could also influence the level of response preparation. For example, as a turn gets longer, probability of its ending gets higher, and this might lead to higher level of preparation for the articulation.

To summarize, studies have shown inconsistent results whether speech planning starts already as soon as the answer is known to questions (Bögels et al., 2015), or it starts only just before the turn-end (Sjerps and Meyer, 2015). It is also little known whether speakers can estimate the duration of turns based on the estimated length of the anticipated linguistic element (e.g., words, phrases) which terminate the turns and whether uncertainty of the estimation of turn-durations influences response times. A confirmation of the effect of lexical anticipation by an experiment with verbal responses could also provide stronger evidence compared to earlier studies using button-press paradigms (De Ruiter et al., 2006; Magyari and De Ruiter, 2012; Magyari et al., 2014). Therefore, we were interested to learn (1) whether speakers could start the speech production process before the last word of the questions; and (2) whether anticipation of the number of syllables of the final word of questions helped in the temporal prediction of the end of questions.

Thus, our experiment was designed to manipulate preparation of what to respond independently of preparation of when to respond. The participants were tasked with answering recorded questions about objects presented on a display. Regarding the first experimental manipulation (preparation of the content of a response), participants could guess the correct answer either (1) early, i.e., toward the beginning of the question or (2) late, i.e., only when they recognized the last word of the question. In the first case, the correct answer to the question could be guessed only by looking at the picture. With respect to the other manipulation (anticipation of the number of syllables of last words of questions), the two possible last words of the questions were either (1) similar in the number of syllables (either both 3–4 syllables long or both monosyllabic) or (2) different (one possible word-candidate had 3–4 syllables, the other was monosyllabic). In the first case, participants could certainly anticipate the length of final word based on the number of syllables of the possible candidates. In the other case, it was uncertain before the recognition of the last word how many syllables of the last word contained. In none of the conditions, participant could guess in advance which word finished the questions.

We reasoned that decreased response times when the answer was known in advance, would suggest that response preparation starts prior to recognition of the last word of the questions. However, if next speakers start to prepare their answer when they recognize the last word, there would be no difference in the response times. If response times increased when it was more uncertain how many syllables the final word of a question had, it would show that word-durations are anticipated and taken into account in response-timing. If next speakers do not predict the duration of words, there would be no differences in response times following this manipulation.

## Materials and Methods

### Subjects

Forty university students between the ages of 18 and 25 years (10 male and 30 female) participated in the experiment. They were registered in the subject pool of the Max Planck Institute for Psycholinguistics in Nijmegen and were paid for their participation. All were native Dutch speakers. At the time of commencement of the study, ethical approval was not required for this study design according to the local legislation.

### Stimuli

**FIGURE 1:**
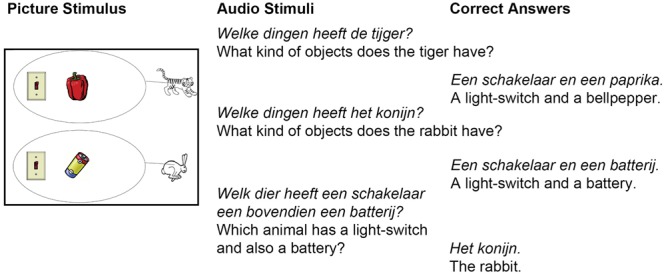
**An example of the stimuli and the intended answer.** An image was displayed on the computer screen (Picture Stimulus, Left) while recordings of the three questions were played (Audio Stimuli, Middle column). The participants’ task was to answer the questions (Correct Answers, Right column). The experiment was in Dutch. The English translation is written in regular letter type.

Participants looked at pictures on a computer screen and heard questions about the presented pictures. On each picture, black and white drawings of two animals, a tiger and a rabbit and two circles with drawings of colorful objects were presented (see an example in **Figure 1**). The drawings were from the picture database of the Max Planck Institute for Psycholinguistics or from free web sources.

The questions and names of objects were recorded in advance by a female native speaker of standard Dutch who was asked to read the questions and names of the objects in a natural speaking rate.

### Experimental Design

An experimental trial consisted of presenting a picture with two scenes (one with a rabbit and one with a tiger above each other) and three questions. Participants were asked to answer the questions while looking at the picture (**Figure 1**). The first two questions were the same in each trial. These questions aimed to control if participants named objects as intended and required participants to observe the picture carefully. These control questions asked which objects each animal had. The tiger and the rabbit were said to “have” the objects in the circle behind them. The third question was the critical question which was different across experimental trials. The critical question always named two objects, and it asked to which animal these objects belonged. The participants’ response time was measured only after this last (critical) question.

**FIGURE 2:**
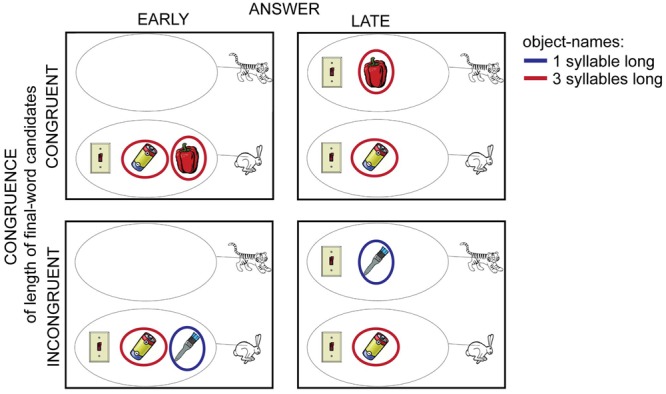
**One set of pictures from the different experimental conditions.** The critical question during these pictures and the right answer was the same: “Which animal has a light-switch and also a battery?”, “The rabbit”. When pictures of the left column were presented (ANSWER: EARLY), participants could already guess the answer at the beginning of the question. When pictures were presented in the upper row (CONGRUENCE: CONGRUENT), participants could guess the length of the final word after listening to the first part of the question, because the names of the remaining objects had the same number of syllables (red circles). In the INCONGRUENT condition (lower row) the potential last words had different number of syllables (red and blue circles). The red and blue circles were not part of the presented images; they serve only the purpose of illustration here.

The same questions were presented to all participants. However, the pictures belonging to the questions were different in the different experimental conditions. The experimental conditions varied in respect to whether participants could guess the correct answer at the beginning of the critical question or only after recognizing the last word of the critical question (ANSWER: EARLY vs. LATE). The left column of **Figure 2** shows two examples of the picture stimuli in the EARLY conditions. When no objects were presented for one of the animals, the participant could be certain that the correct answer for the critical question would be the name of the other animal.

The other experimental manipulation measured if participants could estimate with certainty how long the critical question would take to complete (CONGRUENCE: INCONGRUENT vs. CONGRUENT). There were always three different objects presented in each picture stimulus. Two of these objects were mentioned in the critical question. When the first object was mentioned (e.g., “Welk dier heeft een schakelaar ….” Which animal has a light-switch…), the final word of the question named one of the two remaining objects (e.g., “paprika” or “batterij,” bell-pepper or battery, **Figure 1**). When the name of these two objects had the same length measured in the number of syllables, participants could more certainly predict the duration of the question before hearing the last word (upper row of **Figure 2**) (CONGRUENT conditions). When the number of syllables was different between the possible final words, the prediction was less certain (bottom row of **Figure 2**) (INCONGRUENT conditions). The four experimental conditions were created from these two main experimental manipulations: EARLY-CONGRUENT [e.g., left image in upper row of **Figure 2**, one of the animals had objects and the names of objects which were the possible candidates for the final word of the question had the same number of syllables: “paprika” (bell-pepper) and “batterij” (battery)], LATE-CONGRUENT [e.g., right image in upper row of **Figure 2**, both of the animals had objects and the names of objects which were the possible candidates for the final word of the question had the same number of syllables: “paprika” (bell-pepper) and “batterij” (battery)], EARLY-INCONGRUENT [e.g., left image in bottom row of **Figure 2**, one of the animals had objects and the names of objects which were the possible candidates for the final word of the question had different numbers of syllables: “kwast” (brush) and “batterij” (battery)], LATE-INCONGRUENT [e.g., right image in bottom row of **Figure 2**, both of the animals had objects and the names of objects which were the possible candidates for the final word of the question had different numbers of syllables: “kwast” (brush) and “batterij” (battery)].

Twenty different critical questions were used, of which half ended in a short word (1 syllable long) and the other half in a long word (3–4 syllables long). Similarly, the correct answer was “het konijn” (the rabbit) and “de tijger” (the tiger) 10 times, respectively. Each participant was presented with these 20 critical questions after the two control questions. The order of the critical questions was randomly determined and was the same for each participant. Each of the participants was assigned to one of four experimental lists. While the questions, answers and their order were identical in each list, different versions of a picture were presented with the same question in the different experimental lists. So, the experimental condition of each question varied across lists. Such a set of pictures belonging to one critical question is shown in **Figure 2**. In each experimental list, an equal number of critical questions belonged to each experimental condition. Each list was assigned to 10 participants.

To summarize, participants were presented with the same 20 critical questions in the same order and 2 control questions before each of the critical questions. Each group of participants was assigned differing conditions for each critical question and each condition was assigned an equal number of times. Additional features were balanced across the critical questions: the length of the last words of the critical questions, the type of the answer (“het konijn” or “de tijger”) and the position of the object representing the final word in the visual scenes. A list of the critical questions and the competitors of the final words of these questions can be found in the Supplementary Table 1.

### Procedure

Participants were seated in a soundproof cabin in front of the screen of a computer presenting the experimental stimuli, wearing headphones, and with a microphone and button-box in front of them. Participants’ voices were recorded continuously throughout the experiment using Digital Audio Tape (DAT). Pictures and sound stimuli were presented using the software Presentation 12.1. Button-presses were recorded in log files and on DAT using a beep. The pre-recorded sound stimuli (words and questions) were also recorded when they were played. The sound stimuli and the beep of button presses were recorded on a different track than the participants’ voices.

First, a warm-up session was conducted to familiarize participants with the object-images and their names. This session consisted of each object being displayed along with an audio stimulus of its name. This round was run twice. In a third round, the participants were asked to produce the name of the displayed object. Both written and verbal instructions were provided. The round started when the participant pressed a button. Then, each image was displayed for 3 s in a random order along with the audio of its name. In the third run, a trial started when participants pressed a button. First, a cross appeared for 500 ms. Then, the image appeared on screen, the participant produced the name of the object and pressed a button to initiate the next trial.

After the warm-up session, written and verbal instructions were provided for the second part of the experiment. This part started with five practice trials. These trials were similar to the experimental trials but used different stimulus material. Participants were asked to look at the pictures displayed in front of them and answer the three questions about each picture as quickly as possible after the end of the question. With this instruction, we aimed to avoid a quiz-like situation in which participants would answer as soon as they knew the answer. The instruction also encouraged participants to respond quick after the end of the questions, as one might do in real conversation. A trial began when the participant pressed a button, and the picture stimulus appeared on the screen. After 250 ms, the first question was played. Once the participants answered, they had to press a button to hear the next question. When all three questions for the picture were answered, a new picture appeared on the screen after a button-press.

### Measuring Response

The recordings of the audio stimuli and the participants’ voice were converted to WAV files using Audacity 1.2.6. The files were analyzed with Praat 5.1. Response times were measured as the duration between the end of the critical questions and the beginning of the participant’s answer. Negative response times indicate an overlap between question and answer. The ending of a critical question and the beginning of an answer were manually coded using the intensity wave, the spectrogram, and by ear. The beginning of the first speech-sound (excluding inhalations or hesitations) was marked as the beginning of an answer.

### Statistical Analysis

For statistical evaluation of the results, we used a (linear) mixed-effects model with maximum likelihood estimation. The lme4 package (Bates and Maechler, 2009) of R, an open-source language and environment for statistical computing was used for the statistical analysis (R Development Core Team, 2009). Model-reduction and the selection of the random-effect structure of the mixed-effects analysis were done in three steps. First, the fixed effect-structure was determined. The initial mixed-effects model contained all experimental variables with all their interactions (up to 3-way) and control variables without interactions. Intercepts of subjects and items were included as random effects. This model was reduced step-by-step. Significance of the interactions was evaluated by likelihood-ratio test with a 0.05 alpha-level for model-selection, where the model was compared to a similar model without the interaction of interest. In a second step, the reduced model was extended with the slopes and interactions of the fixed effects in the random effect structure. If the model with all random effects did not converge, a forward “best path” algorithm was used to evaluate which random slope (and interaction) should be included. *P*-values were derived from a likelihood-ratio test with a 0.4 alpha-level for model-selection (Barr et al., 2013). At the end of this second step, we reached a model which contained fixed effects from the first step and an extended random effect structure. In third step, the significance of the fixed effects of this model was once again evaluated with model comparison by likelihood-ratio tests. The model was compared to a similar model without the fixed effect of interest. If any interaction between fixed-effects was no longer significant, it was removed from the model. The mixed-effects model was run with a maximum of 1000 iterations.

We analyzed the effect of the control and the experimental variables on the response times. Control variables were variables which could affect response times but were not consistently manipulated in the experiment. These variables were (1) age of the participants (AGE), (2) gender of the participants (GENDER), (3) the order of stimulus presentation (ORDER), (4) the experimental list (LIST), (5) whether the answer contained a determiner (“het” or “de”; DET), (6) whether the answer was “het konijn”/“konijn” or “de tijger”/“tijger” (ANSWER_TYPE). Experimental variables were variables which were manipulated consistently in the experimental design. ANSWER was a factor with two levels which showed whether participants could know the answer for the critical questions early or late, CONGRUENCE was a factor with two levels which coded whether the candidate final words of the critical question were the same or different in length and FINAL_WORD was a continuous variable. FINAL_WORD coded the duration of the last words in milliseconds. It was included because the questions differed in the length of the last words as a result of the experimental design. All continuous variables were centered.

## Results

### Analysis of Response Times

**FIGURE 3:**
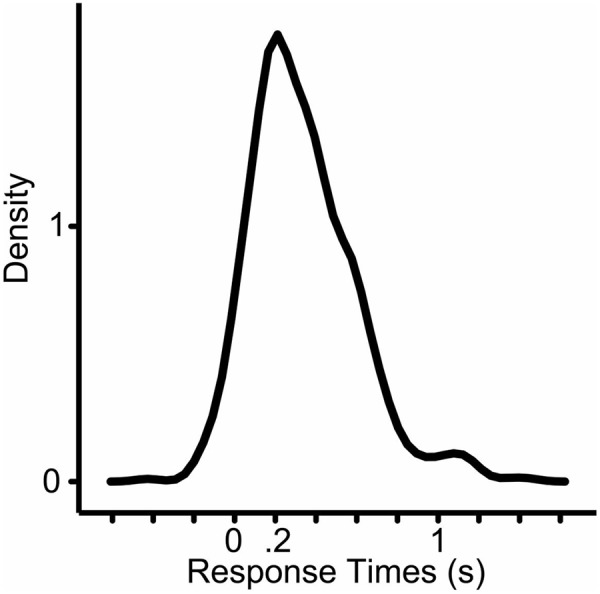
**Density plot of the analyzed response times (*N* = 620).** *X*-axis shows the response times in seconds.

One participant’s data was excluded from the statistical analysis because of remarkably different responses compared to all other subjects (average response time of this subject were more than two standard deviations shorter than the mean of all responses). Answers that were wrong, contained hesitations, false starts, laughter were excluded. Answers were also excluded if participants already pressed the button for the next trial while they were answering the critical question. The analyzed data had 620 data-points (mean = 336 ms, median = 299 ms, *SD* = 258, min = -427 ms, max = 1442 ms; 22,5% of all data was excluded) (**Figure 3**).

In our mixed-effects model, the response variable was the response time, measured as the duration from the turn end to the beginning of the answer. Fixed effects were whether participants could know the answer early or late (ANSWER), whether the competitor of the final word was the similar or different in length (CONGRUENCE), the duration of the final word of the critical question as a continuous variable (FINAL_WORD) and the control variables (AGE, GENDER, LIST, DET, ANSWER_TYPE). The experimental variables participated in the initial model with all of their interactions; the control variables were included as single main effects. Subjects and items were random effects. After model simplification and the expansion of the random effects, the final model contained the main effect of all variables and the interaction of CONGRUENCE and FINAL_WORD. Subjects, items and the slope of ANSWER under subjects were the random effects in the final model. **Table 1** summarizes the results.

**Table 1:** Beta-coefficients, chi-square, and *p*-values of the fixed effects in the final mixed-effects model.

Variable	Level (if categorical)	β	χ^2^(1)	*p*
ANSWER	Late	0.046	5.51	**0.019**
Duration of FINAL_WORD		-0.38	28.04	**<0.0001**
CONGRUENCE	Incongruent	0.01	4.29	0.117
GENDER	Female	0.07	1.32	0.25
Experimental LIST	2	-0.04	0.57	0.904
Experimental LIST	3	-0.02		
Experimental LIST	4	0.01		
ORDER of stimulus presentation		0.0005	0.08	0.771
DET	Article was used	-0.004	0.015	0.903
ANSWER_TYPE	“De Tijger.”	0.006	0.084	0.772
FINAL_WORD^∗^ CONGRUENCE	Incongruent	-0.24	4.168	**0.041**

*Chi-square and *p*-values were obtained by model-comparison. The ^∗^ denotes interaction, *p*-values of significant effects are written with bold letter type.*

**FIGURE 4:**
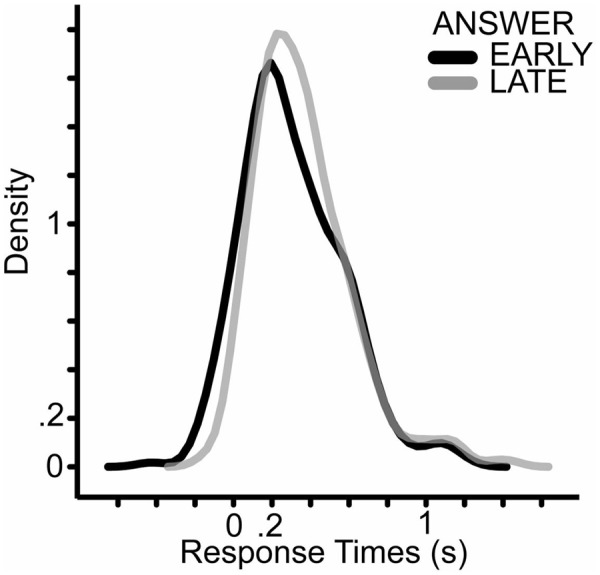
**Density plot of response times in the ANSWER conditions.** The dark line shows the density plot of responses when participants knew the answer for the questions early; the light gray line shows the responses when the participants could guess the answer only at the last word of the question.

ANSWER, FINAL_WORD and the interaction of FINAL_WORD and CONGRUENCE showed significant effect on the response times. When the answer was known earlier, participant answered earlier (**Figure 4**). In the EARLY ANSWER condition the mean of response times was 320 ms and the median was 269 ms, in the LATE ANSWER conditions the mean of the response time was 361 ms and the median was 314 ms.

The fastest response in the EARLY condition was around – 400 ms while the shortest question was 3 s long. In the EARLY condition participants could guess the right answer for the critical question already at the beginning of the question. Therefore, it is unlikely that there was an effect of ANSWER because participants answered as soon as they knew the answer to the questions. However, the effect of ANSWER shows that participants started to prepare their answer before the end or even before the last word of the question in the EARLY conditions.

**FIGURE 5:**
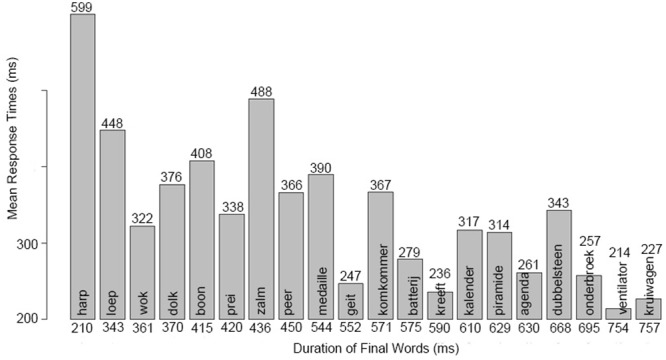
**Mean response times for each critical question.** The *y*-axis shows the average of the response times in ms. The mean response times are plotted in increasing order of the final word-duration of the critical questions on the *x*-axis. The final word of each question is written on the bars, the mean response times are shown on the top, and the durations of the final words are presented at the bottom of the bars. Response times are faster when the final words are longer.

The durations of the final words also affected response times significantly. When the words were shorter, participants answered later (**Figure 5**).

**FIGURE 6:**
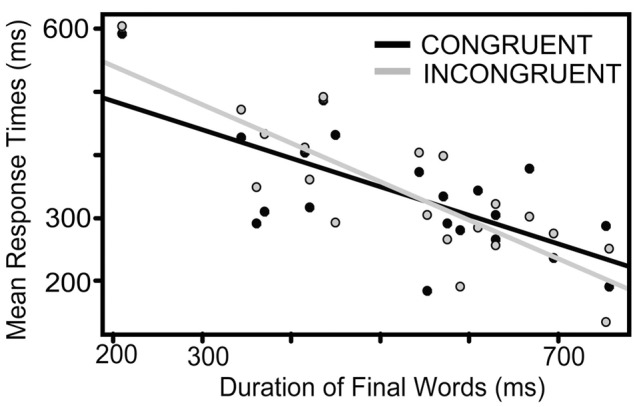
**The average of the response times as a function of the duration of the final words in the congruent (black) and in the incongruent conditions (gray).** The final word-durations are presented on the *x*-axis, mean response times on the *y*-axis. The gray and black line shows the regression line between mean response times and final word duration in the congruent (black) and in the incongruent (gray) conditions.

The interaction between the duration of the final words and CONGRUENCE shows that the duration of the competitor word affected the response differently for shorter and longer final words. **Figure 6** shows the mean response times as a function of final word durations in the congruent and incongruent conditions; and the regression lines, respectively. In the congruent condition the number of syllables of the final word and the competitor was the same, in the incongruent condition the number of syllables were different. Our initial hypothesis was that uncertainty about the duration of the critical question (incongruent condition) leads to less preparation which results in longer response times. This has been not confirmed, because CONGRUENCE did not have a significant main effect. However, **Figure 6** shows that response time increased in the incongruent condition when the final words were shorter.

**FIGURE 7:**
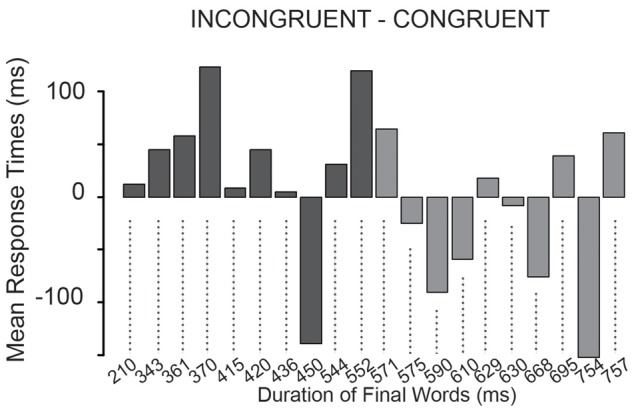
**The difference of the mean response times between the incongruent and congruent condition for each critical question.** *Y*-axis shows the response times differences in ms, the *x*-axis shows the critical questions in increasing order of the duration of their final words from left to right.

**Figure 7** shows the differences of the mean response times in the congruent and in the incongruent conditions for each critical question in the order of the durations of their final words.

In a *post hoc* analysis, we computed Wilcoxon signed-rank test on the mean difference of response times between the congruent and incongruent conditions separately for the critical questions with the 10 shortest (**Figure 7**, dark gray) and with the other 10 final words (light gray). We tested whether the difference between conditions was different from 0. For the shortest final words, there was a trend in the deviation from 0 mean (*V* = 45, *p* = 0.084), while there was no trend or significant difference for the other group (*V* = 19, *p* = 0.43). This might suggest that uncertainty of the final word duration increased response times only when the final words had a short duration.

### Analysis of Response Times Relative to Phonetic and Lexical Cues

In an additional analysis, we checked whether participants’ reaction to phonetic or lexical cues could lead to the effect of final word duration. It was possible that participants started the production of their answers as a reaction to such cues. For example, if cues are closer to the end of questions with shorter final words, it could explain why the response times vary as a function of final word duration. However, if the effect of final word duration cannot only be explained by reaction to cues, other mechanisms for explanation can also be proposed. We checked whether cues cause the effect of final word duration on the response times by using two aspects of this process: First, if speakers start the production of their answer by encountering a turn-yielding cue, and this cue is closer to the turn-end in shorter final words, responses will be produced later to questions ending with short words. To examine this assumption, we checked whether there were correlations between the durations of the final words and the durations from possible cues to the end of the questions (i.e., end of the last word of the questions). Second, if speakers start the production of their answer as a reaction to a cue, response latencies relative to the given cue will be not affected by the rest of the question measured from the cue to the end of the last word. For this, we calculated the response latencies relative to the possible cues and used mixed-effect modeling to reveal their relationship with the duration of the rest of the question.

One of the most obvious cue to which speakers could time-lock the speech preparation process or the articulation is the beginning of the last word. If the final word of a question is longer, there is more time for speech production, so speakers can answer to questions faster relative to the end of the question. However, there might be also other potential turn-yielding cues in the questions which could help in timing speech production. We examined the effect of the following features in the questions: beginning of the final word, beginning of the question, beginning of the vowel in the final stressed syllable, peak of the final rise in pitch and uniqueness point of the final word. The uniqueness point of a word is the moment during listening to a word when only one real word is consistent with all of the input received up to that point. We examined the effect of this point because word recognition can take place sooner if a word has an earlier lexical uniqueness point (Samuel, 2011).

First, we tested whether there was a correlation between the durations of the final words and the durations from the different possible cues to the end of the last word. If there are cues which are closer to word-end in shorter final words, these cues are potential candidates for explaining the effect of final word durations on the response times in the main analysis. The duration of the questions [*r*(18) = 0.851, *p* < 0.001] and the duration between the uniqueness point and the word-end [*r*(18) = 0.62, *p* = 0.004] showed significant correlation with the final word duration. The correlation was not significant with the other two features [final pitch: *r*(18) = -0.13, *p* = 0.6; final stress: *r*(18) = 0.38; *p* = 0.094].

Then, we calculated response latencies from these possible cues (from the uniqueness point, from the beginning of the questions) and from the beginning of the final word of the questions to the beginning of the answer. We evaluated whether the new response latencies are influenced by the durations between the cues and the end of the last words. If there was an effect, this could not be explained purely by reaction to cues. In contrast, if a response was initiated purely as a reaction to a cue, the duration of the rest of the question relative to the cue did not have any effect on the new response time. We also included into our analysis the duration between the beginning of the question and the cue as another independent variable. If participants reacted to a cue to initiate the response, temporal expectations and preparation could still occur during the question prior to the cue. If this was the case, the later a cue appeared in the questions, more prepared speakers were to respond. More preparation could lead to faster responses. However, this effect should be still independent from the duration of the rest of the turn after a cue if speakers time their production to a cue.

Separate mixed-effects models were run with the newly calculated response times, respectively, for the uniqueness point, the beginning of the final word and the beginning of the questions. In the first two models the duration of the questions before and after the cues were included as fixed effects. Before applying the regression model, it was checked if there were correlations between the durations before and after these cues. There were no significant correlations [uniqueness point: *r* = 0.028, *p*(18) = 0.907; beginning of final word: *r*(18) = 0.19, *p* = 0.418].

When the beginning of the question was tested as the potential cue, the duration of the question was included as the only fixed effect in the model. We followed the statistical modeling procedure described in Section “Statistical Analysis.” **Table 2** summarizes the results.

**Table 2:** Beta-coefficients (β), chi-squares (χ^2^), and *p*-values of the fixed effects (duration from the beginning of the question to cue and duration from cue to end of question) in three mixed-effect models are listed by row.

Cue	Duration from beginning of question to cue			Duration from cue to end of question		
	β	χ^2^(1)	*p*	β	χ^2^(1)	*p*
UP	-0.28	10.5	**<0.01**	0.48	11.76	**<0.001**
FW	-0.07	0.59	0.444	0.49	22.15	**<0.0001**
Q	n.a.	n.a.	n.a.	0.65	34.47	**<0.0001**

*Chi-square and *p*-values were obtained by model-comparison (see Statistical Analysis; Bonferroni-corrected significance level: 0.016). The cues which were used for measuring the fixed effects and the response variables are abbreviated as follows: UP – uniqueness point, FW – the beginning of the final word, Q – the beginning of the question. *P*-values of significant effects are written in bold letter type.*

When response times were calculated from the uniqueness point of the final word of questions, the duration of questions before the uniqueness point and the duration after the uniqueness point have affected the response times. As the duration before the uniqueness point increased, the response time decreased. However, when the duration of the question after the uniqueness point was longer, response time increased. When the response time was calculated relative to the beginning of the last word, the duration of the question before the last word did not show a significant effect on the response time. But the duration of the question after the beginning of the last word, i.e., the duration of the final word positively correlated with the response time. When response times were calculated relative to the beginning of questions, response times significantly increased at longer questions. In sum, all analysis showed that all type of response time positively correlated with the duration between the potential cues at the end of the questions.

These analyses showed that none of the examined features (beginning of the question, beginning of the final word and the uniqueness point of the final word) served as the only cue for starting speech production. It is still possible that these features helped in recognizing when the question was going to end and when speech production could start. However, the position of cues relative to turn-ends cannot alone explain the decrease in response times when the final words were shorter in our earlier analysis (see Analysis of Response Times). Hence, it is likely that the expected duration of the final words influenced response times.

## Discussion

We were interested to know more about how interactants are able to achieve fast transitions from listener’s to speaker’s role during turn-taking. In our experiment, we examined whether speakers started the speech production process before the terminal element, i.e., before the last word of questions when they already had the possibility to guess a correct answer; and (2) whether they anticipated the length of final word of questions to predict durations.

In the experiment, we measured participants’ response times when they answered pre-recorded questions about images presented on a screen. Response time was measured between the question end and the beginning of the answer. We varied in different experimental conditions whether people knew the correct answer early or late and whether it was possible to predict with certainty the number of syllables of the words which ended the questions. The level of certainty was manipulated by presenting questions that could end in the names of one of two objects in the presented image. The objects were chosen so their names either have the same (congruent condition) or different number of syllables (incongruent condition). The questions also differed in duration of their final words.

The results confirmed one of our predictions. When participants could only prepare the answer late, their response times were longer. Our second prediction was that participants answer later when they are uncertain about the duration of the question. There was no significant main effect of the certainty-manipulation, but there was an interaction effect between congruence and the duration of the final words. When the final words of questions were shorter, response times were longer in the incongruent conditions in which participants could not predict the length of the final words. The duration of the final words also had a main effect. When the last words were longer, response times decreased.

When participants already knew the answer at the beginning of the questions, they had responded in average 310 ms after the question end. This shows that participants did not answer as soon as they knew the answer but were waiting for the right moment to start to speak. This is not surprising because we explicitly instructed participants to respond quickly after the question ended. The difference in response latencies in the early and late answer conditions also shows that participants started speech production before the recognition of the last word of the questions in the early condition. The results confirm that next speakers in a conversation will prepare their turn earlier if they know what to respond. This finding is in line with a study of Bögels et al. (2015) using a quiz-game paradigm. The study has shown that participants produced shorter turn-transitions answering questions when they were able to guess the answer to a question before its final word (Bögels et al., 2015).

When the last words were longer, participants answered faster. One explanation of this effect could be that participants answered in a fixed time interval relative to a feature in the last words, for example, the beginning of the last word, the uniqueness point, the final pitch or the final stressed syllable. If any of these features occurs earlier relative to question end in longer final words, and participants time the start of the production of their answer to this point, it could explain the shorter gap after longer words. However, in our additional analysis we found that none of these cues alone could explain the difference in response times according to final word duration. Therefore, it is likely that the expected final word durations influenced response time based on the probability distribution of final word durations throughout the experiment. In interval-timing experiments when the momentary probability of the end of an interval is higher, reaction times decrease (Niemi and Näätänen, 1981). In our experiment, as the duration of the final word continued to grow, the probability of its ending got higher (see Supplementary Figure 1). Hence, the level of response preparation also increased with the duration of the final words, which is reflected in the response times.

The significant interaction between congruence and final word durations suggests that temporal expectations independent of duration probabilities also affected the response times. For the critical questions with shorter final words, response times were longer when the length of the competitor was incongruent. However, this was only marginally confirmed in the *post hoc* analysis. Therefore, the interpretation of the results should be handled carefully. The interaction might suggest that there is a difference in the process of preparation when the time-interval until the turn-end is estimated to be shorter or longer. Such explanation corresponds to conclusion of studies where a symbolic cue predicts the duration of the interval between a warning and a response signal. When there is a mismatch between the symbolic cue and the actual interval duration, reaction times increase. However, when the duration between the warning and response signal is longer, reaction times are less affected by the mismatch. This is explained by re-orientation of temporal attention. When a response signal is expected to appear early but it appears late, participants still have time to focus their attention on the later time-point (Coull and Nobre, 1998; Capizzi et al., 2012). In our experiment, when the final word did not end early (i.e., it was long), participants still had enough time to prepare for a later ending independently of our uncertainty manipulation. In non-linguistic tasks, temporal expectations which are based on symbolic features of a warning signal are found to be dependent on the attention toward the feature (Rohenkohl et al., 2011) and on the working-memory load (Capizzi et al., 2012). Therefore, it is a question for further research whether temporal expectations based on the length of predicted words are attenuated when listeners are facing demanding parallel tasks, for example, difficulties in comprehension or production. It could be especially interesting to know how these processes are affected by efforts of speakers of a second language.

## Conclusion

**FIGURE 8:**
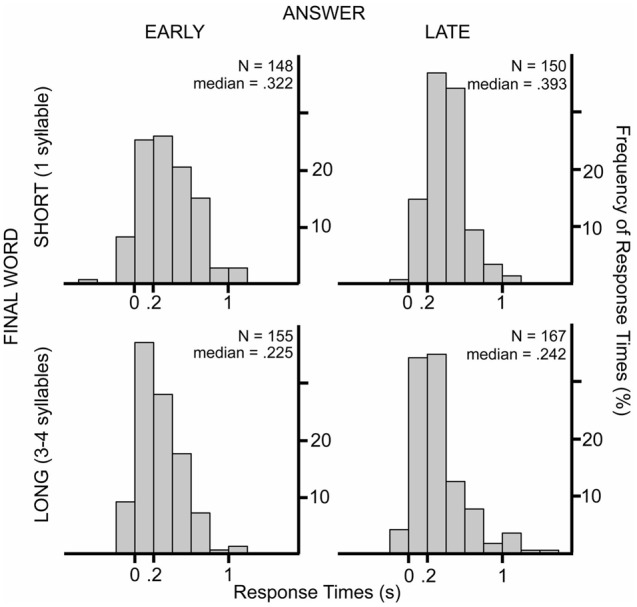
**Histograms show the frequency of response times in 200 ms bins in the different experimental conditions.** The upper panels show response times when the final words were monosyllabic (SHORT); the lower panels show response times when the final words were polysyllabic (LONG). The response times presented in the left panels were produced when the answer was known at the beginning of the questions (EARLY), the right panels show results when the answer could be known only at the last word (LATE).

Our results suggests that participants prepared the production of their answers before the questions ended and tried to time their production to the end of the questions, yet there was still a substantial gap between the end of the questions and the beginning of the answers. The average response latency was 336 ms with a mode between 200 and 400 ms (median = 299 ms) (see **Figure 3**). This is longer than the average duration and mode of turn-transition times reported at answers to polar question in Dutch face-to-face conversations (mean = 108 ms, mode = 100; Stivers et al., 2009) or at any type of turns of Dutch telephone-like conversations (De Ruiter et al., 2006). This difference is not surprising because the question-answer sequences of our experiments are far from a natural, conversational setting. Moreover, answers which contained hesitations, false starts or laughs were excluded from our analysis. In every-day conversations, a turn may start with hesitations before the actual answer. **Figure 8** shows the frequency of the response times when responses are grouped according to length of the final words (short: monosyllabic or long: polysyllabic) and when the answer was already known at beginning of the questions or was known later (at the recognition of the last word of a question).

The medians of response times were around 200 ms at turns with longer final words. When the turns ended with a monosyllabic word the medians were larger than 300 ms even if participants knew the answer already from the beginning of the question. Therefore, we conclude that the most frequent turn-transition times (0–200 ms) of every-day conversations can be only produced when speakers are prepared for the turn-end already before the last syllable.

To summarize, we showed that speakers will start to prepare for the production of their answer before the current turn ends and they also prepare for the time to speak. This preparation can be influenced by linguistic and non-linguistic temporal expectations. Non-linguistic temporal expectations can arise from the overall distribution of turn durations. With other words, addressees know that they will need to respond and as the turn continues, the probability of its end increases, so the level of preparation for speaking will also increase. This preparation can occur even before addressees would know what to respond. For example, the neuronal network of speech production can be already set up before the actual content of the to-be-produced speech is retrieved (Gehrig et al., 2012). Furthermore, temporal predictions are not restricted to motor behavior. Temporal expectations can also modulate attention and perceptual processes (Nobre et al., 2007) which could facilitate the recognition of terminal elements of turns.

## Ethics Statement

The study was conducted at the Max Planck Institute for Psycholinguistics. Before the experiment, the study was presented and approved by the research staff of the Department of Language and Cognition with attention to ethical considerations in compliance with the Declaration of Helsinki. At the time of commencement of the study, ethical approval was not required for this study design according to the local legislation.

Participants were recruited from the subject pool of the Max Planck Institute for Psycholinguistics via email and were paid for their participation. The email has already contained a description of the experimental task. When participants arrived to the experiment, their task was verbally explained to them again, and they only started the experiment when they agreed to participate.

Healthy participants between 18 and 25 years participated in the study, therefore, additional considerations are not applicable.

## Author Contributions

LM, JD, and SL have designed the study, interpreted the results and written the article. LM has conducted the experiment and analysed the data.

## Conflict of Interest Statement

The authors declare that the research was conducted in the absence of any commercial or financial relationships that could be construed as a potential conflict of interest.
