# Supplementary material for: Temporal Preparation for Speaking in Question-Answer Sequences
Source: Front Psychol. 2017 Feb 21;8:211. doi: 10.3389/fpsyg.2017.00211 (PMC5318421; doi:10.3389/fpsyg.2017.00211)

Supplementary Material

Temporal Preparation for Speaking in Question-Answer Sequences

Lilla Magyari*, Jan P. De Ruiter, Stephen C. Levinson

*** Correspondence:** Lilla Magyari: magyari.lilla@btk.ppke.hu

1 Supplementary Tables

Supplementary Table 1. The critical questions and the competitors of their final words

Critical Questions	*Competitors of the final words*	
	Short	*Long*
Welk dier heeft een casette en bovendien ook een loep?	spons	antenne
Welk dier heeft een magnetron en bovendien ook een wok?	stronk	kandelaar
Welk dier heeft een lucifer en bovendien ook een kruiwagen?	pauw	kruiwagen
Welk dier heeft een kastanje en bovendien ook een Piramide?	schaar	verrekijker
Welk dier heeft een torpedo en bovendien ook een dobbelsteen?	vlecht	aansteker
Welk dier heeft een brievenbus en bovendien ook een geit?	kruk	paddestoel
Welk dier heeft een liniaal en bovendien ook een harp?	zeis	ananas
Welk dier heeft een boterham en bovendien ook een onderbroek?	taart	boterham
Welk dier heeft een accordeon en bovendien ook een Kalender?	spin	paraplu
Welk dier heeft een zonnebril en bovendien ook een Medaille?	kam	diamant
Welk dier heeft een sigaret en bovendien ook een boon?	kaars	piano
Welk dier heeft een schakelaar en bovendien ook een batterij?	kwast	paprika
Welk dier heeft een portemonaie en bovendien ook een komkommer?	fluit	microscoop
Welk dier heeft een computer en bovendien ook een		

peer?	sjaal	helikopter
Welk dier heeft een aubergine en bovendien ook een prei?	tol	bikini
Welk dier heeft een asperge en bovendien ook een zalm?	kurk	pantoffel
Welk dier heeft een telefoon en bovendien ook een agenda?	bijl	envelope
Welk dier heeft een capuchon en bovendien ook een ventilator?	zaag	thermometer
Welk dier heeft een camera en bovendien ook een dolk?	kers	parasol
Welk dier heeft een stofzuiger en bovendien ook een kreeft?	tang	parachute

2 Supplementary Figures

Supplementary Figure 1. The upper panel shows the frequency of the final word-durations in 100 ms bins in our experimental stimuli. The lower panel shows the momentary probability of the ending of final words (expectancy) in the next 100 ms based on the frequencies in the upper panel.

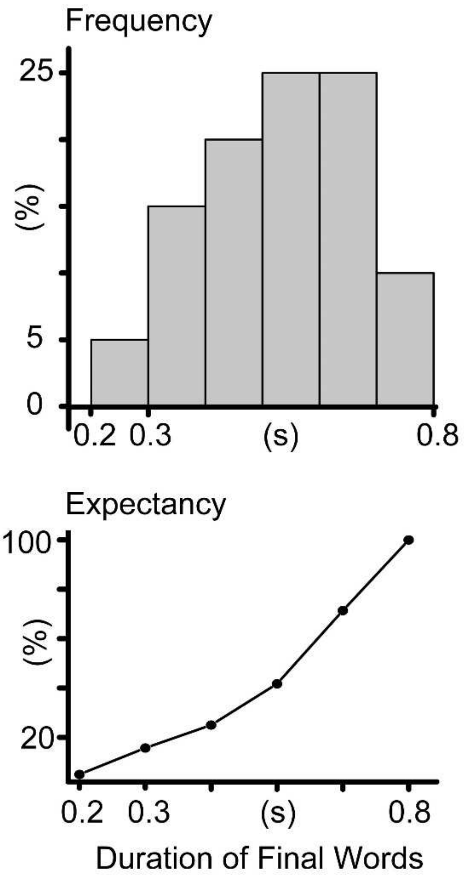

Supplement: Supplementary file 1 [file Presentation_1.pdf]
